# Comprehensive analyses of long non-coding RNA expression profiles by RNA sequencing and exploration of their potency as biomarkers in psoriatic arthritis patients

**DOI:** 10.1186/s12865-019-0297-9

**Published:** 2019-08-07

**Authors:** Tao Yue, Mei Ji, Huanru Qu, Mengru Guo, Fengmin Bai, Zhanming Zhang, Weifeng Wang, Xuming Gong, Zhenghua Zhang

**Affiliations:** 1 Department of Rheumatology, Shanghai Guanghua Hospital of Integrated Traditional Chinese and Western Medicine, 540 Xinhua Road, Shanghai, 200052 China; 2 Department of Dermatology, Skin Disease Prevention and Treatment of Fengxian District of Shanghai, Shanghai, China; 3 Department of Rheumatology, Longhua Hospital affiliated to Shanghai University of Traditional Chinese Medicine, Shanghai, China; 4 Department of Dermatology, Huashan Hospital, Fudan University, 12 Wulumuqi Zhong Road, Shanghai, 200040 China

**Keywords:** Psoriatic arthritis, Long non-coding RNA, Expression profiles, RNA sequencing, Biomarkers

## Abstract

**Background:**

The aim of the current study was to investigate the long non-coding RNA (lncRNA) expression profiles in psoriatic arthritis (PSA) patients by RNA sequencing, and to further explore potential biomarkers that were able to predict PSA risk and activity.

**Methods:**

LncRNA and mRNA expression profiles in peripheral blood mononuclear cells (PBMC) of 4 PSA patients and 4 normal controls (NCs) were detected by RNA sequencing, followed by comprehensive bioinformatic analyses. Subsequently, 3 top upregulated and 2 top downregulated lncRNAs were chosen for further validation in 93 PSA patients and 93 NCs by quantitative polymerase chain reaction (qPCR) assay.

**Results:**

Totally 76 upregulated and 54 downregulated lncRNAs, as well as 231 upregulated and 102 downregulated mRNAs were discovered in PSA patients compared with NCs. Enrichment analyses revealed that they were mostly associated with nucleosome, extracellular exosome and extracellular matrix, and the top enriched pathways were systemic lupus erythematosus (SLE), alcoholism and viral carcinogenesis. qPCR assay showed that lnc-RP11-701H24.7 and lnc-RNU12 were upregulated in PSA patients compared with NCs, and they could predict PSA risk with high area under curves. Besides, lnc-RP11-701H24.7 was positively associated with ESR, SJC, TJC and pain VAS score while lnc-RNU12 was positively correlated with PASI score, CRP and PGA score, implying that both of them were positively correlated with disease activity.

**Conclusion:**

Our study facilitates comprehensive understanding of lncRNA expression profiles in PSA pathogenesis, and discovers that lnc-RP11-701H24.7 and lnc-RNU12 might be served as novel biomarkers for PSA risk and activity.

**Electronic supplementary material:**

The online version of this article (10.1186/s12865-019-0297-9) contains supplementary material, which is available to authorized users.

## Background

Psoriatic arthritis (PSA), a complicated and heterogeneous autoimmune disease that causes severe joint damage and reduces quality of life, is characterized by different symptoms such as enthesitis, dactylitis, nail dystrophy, uveitis, osteitis and several comorbidities including cardiovascular disease (CAD), obesity and metabolic syndrome [1, 2]. It’s reported that approximately 30% of psoriatic patients would occur PSA in their lifetime and there is no “golden standard” diagnostic test for PSA in clinical practices so far, thus the diagnosis that only relies on various pieces of evidence remains a challenge [2–6]. More importantly, the detailed mechanisms underlying pathogenesis of PSA are still poorly illuminated, which greatly obstruct the improvement of long-term outcome of PSA patients [2].

Long non-coding RNA (lncRNA), a class of RNAs that have more than 200 nucleotides and lack open reading frames, is implicated different cancers, CAD and autoimmune diseases [7, 8]. In the past few decades, lncRNA expression profiles of autoimmune diseases such as systemic lupus erythematosus (SLE), rheumatic arthritis (RA) and inflammatory bowel disease (IBD) have been investigated via microarray or RNA sequencing, through which the roles of lncRNA expression profiles in etiology of several autoimmune diseases have been disclosed and a few lncRNAs that present with predicting values for disease risk, inflammation level and activity have been discovered [9–11]. For instance, an interesting study investigates the lncRNA expression profiles in T cells of SLE patients using microarray, which finds 1935 differentially expressed lncRNAs (DELs) in SLE patients compared with healthy controls (HCs), further analyses reveal that both uc001ykl.1 and ENST00000448942 expressions could differentiate SLE patients from controls and positively correlate with disease activity as well as inflammation level [9]. In another study, lncRNA expression profiles in peripheral blood mononuclear cells (PBMC) of RA patients are investigated, then 5045 DELs are identified in RA patients compared with HCs, enrichment analyses indicate that these DELs are mostly implicated bile secretion, T cell receptor signaling pathways and SLE [10].

Considering that PSA is also a polygenic autoimmune disease, exploring lncRNA expression profiles of PSA might also contribute to unveiling development and progression of PSA and discovering biomarkers for predicting PSA risk and activity. However, just one study explores the lncRNA expression profiles in PBMC of PSA patients by microarray, and the DELs in this study are only validated in 20 PSA patients and 20 controls by quantitative polymerase chain reaction (qPCR) assay [12]. Thus, the in-depth knowledge of lncRNA expression profile in PSA pathogenesis remains to be further investigated, and lncRNAs that are able to predict PSA risk and disease activity remain to be discovered. Therefore, the objective of the current study was to explore lncRNA expression profiles in PBMC of PSA patients by RNA sequencing, and to further explore biomarkers (from DELs of PSA) that were able to predict PSA risk and activity.

## Materials and methods

### Patients and samples

Ninety-three PSA patients admitted in Shanghai Guanghua Hospital of Integrated Traditional Chinese and Western Medicine between 2016/7/1 and 2017/6/30 were consecutively enrolled in this study. The inclusion criteria of PSA patients were as follows: (1) Diagnosed as PSA according to the Classification of Psoriatic Arthritis (CASPAR) criteria. (2) Age above 18 years. While patients with the following conditions were excluded: (1) Suffered from other rheumatologic diseases. (2) Complicated with severe heart, kidney or liver diseases. (3) History of severe systemic disease, solid cancers or hematological malignancies. In the meanwhile, 93 healthy volunteers were also recruited and served as normal controls (NCs). After the enrollment, blood samples were obtained from PSA patients and NCs, and then PBMC samples were isolated and stored in liquid nitrogen for further detection.

### Study design and ethics approval

**Fig. 1:**
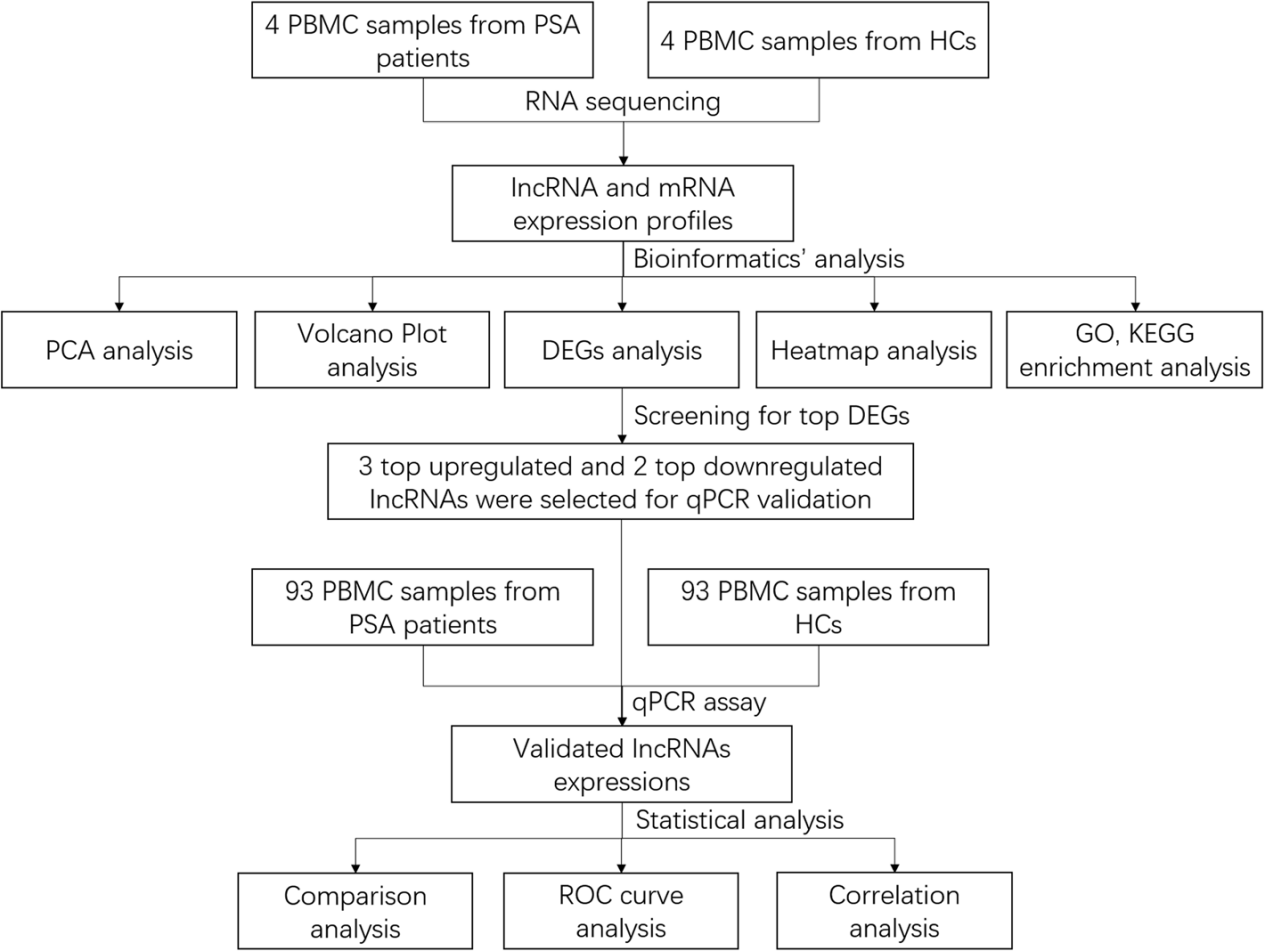
Study flow

The detailed study design was exhibited in Fig. 1. In brief, 4 PBMC samples from PSA patients and 4 PBMC samples from NCs were used for RNA sequencing to obtain lncRNA/mRNA expression profiles, and then comprehensive bioinformatic analyses were performed. Subsequently, 3 top upregulated and 2 top downregulated lncRNAs in PSA were chosen for further validation in 93 PBMC samples from PSA patients and 93 PBMC samples from NCs by qPCR assay. This study was approved by the Ethics Review Board of Shanghai Guanghua Hospital of Integrated Traditional Chinese and Western Medicine, and all patients and NCs signed informed consents.

### Library generation

Total RNA was firstly extracted from 4 PBMC samples from PSA patients and 4 PBMC samples from NCs using Trizol reagent (Invitrogen, USA), and then concentration, purity and integrity were assessed using Qubit® RNA Assay Kit with Qubit® 2.0 Flurometer (Life Technologies, USA), NanoPhotometer® spectrophotometer (IMPLEN, USA) and RNA Nano 6000 Assay Kit with Bioanalyzer 2100 system (Agilent Technologies, USA), respectively. Subsequently, ribosomal RNA (rRNA) was removed from RNA using Epicentre Ribo-zero™ rRNA Removal Kit (Epicentre, USA), and residual RNA was proposed to generate libraries using NEBNext® Ultra™ Directional RNA Library Prep Kit (NEB, USA). Then, after synthetization of first and second strand of the cDNA, library fragments was purified by AMPure XP system (Beckman Coulter, USA) and DNA fragments with a length of 150–200 bp were selected, and then PCR assay was performed. Quality of the library was assessed using Bioanalyzer 2100 system (Agilent Technologies, USA). Then, clustering of index-coded samples was performed using HiSeq PE Cluster Kit v4 cBot (Illumina, USA), and the libraries were sequenced on Illumina Hiseq X10 platform (Illumina, USA), and 150 bp paired-end reads were produced after cluster generation.

### RNA sequencing

Automate quality control and adapter trimming were conducted using Trim Galore, Cutadapt and FastQC. The trimmed reads were mapped to the human genome Hg38 by HISAT2 with default parameters according to the methods in a previous report [13], and mapping quality control was performed using RSeQC referring to the methods in another previous paper [14]. The read counts of lncRNA and mRNA were then calculated using featureCounts based on the annotation file (Homo_sapiens.GRCh38.83.gtf) in Ensembl database according to the methods described in a previous report [15]. In this study, lncRNAs and mRNAs discovered in less than 50% samples were wiped off for analysis, only the remaining lncRNAs and mRNAs were used for follow-up analysis, and their raw reads counts were normalized, logarithmic transformation was applied, and DELs and differentially expressed mRNAs (DEMs) were detected using DeSeq2 as method that was previous described [16], and the statistical significance was defined as adjusted *P* value < 0.05 (Benjamini & Hochberg (BH) adjusted method was applied to reduce the false positive result), and the biological significance was defined as a difference of at least 2.0 folds, which meant abs (log_2_ (fold change)) > =1.0.

### Bioinformatic analyses

Principal component analysis (PCA) of lncRNA and mRNA expression patterns was performed using Stats package. Heatmap plot analysis of total expression pattern, DELs and DEMs were performed using Pheatmap package. Gene Ontology (GO) and Kyoko Encyclopedia of Genes and Genomes (KEGG) enrichment analysis of DELs were performed using DAVID web servers referring to the method in a previous report [17]. Regulatory networks of DELs by cis targets and trans targets were drawn using igraph package. Cis targets of lncRNAs were determined by the location and Trans targets of lncRNAs were determined by pearson correlation coefficient of expressing pattern. All the bioinformatics analysis was performed using R software (Version 3.3.3).

### Data collection

Comprehensive data of PSA patients were collected including age, gender, family history of PSA, disease duration of psoriasis, disease duration of arthritis, psoriasis subtype, arthritis subtype, affected body surface area (BSA), psoriasis area and severity index (PASI) score, C-reactive protein (CRP), erythrocyte sedimentation rate (ESR), swollen joint count (SJC), tender joint count (TJC), Pain visual analogue scale (VAS) score and patients’ global assessment (PGA) score.

### qPCR assay

Total RNAs were extracted from PBMC samples of 93 PSA patients and 93 NCs using Trizol reagent (Invitrogen, USA) according to manufacturer’s instructions, Qubit® 2.0 Flurometer (Life Technologies, USA) was applied for examination of RNA concentration and purity. Subsequently, cDNA was synthesized by QuantiNova Reverse Transcription Kit (Qiagen, German) then it was subjected to qPCR with SYBR Green kit (TaKaRa, Japan). The protocol of qPCR included a single cycle at 95 °C for 5 mins, followed by 40 cycles at 95 °C for 10 s, and annealing at 60 °C for 30 s. After that, the expressions of 5 selected lncRNAs were calculated using the 2^-ΔΔCt^ methods with phosphoglyceraldehyde dehydrogenase (GAPDH) as internal reference. Primers used in qPCR validation were listed in Additional file 1: Table S1.

### Statistics

Statistical analysis was conducted using SPSS 22.0 software (IBM Corp, USA) and GraphPad Prism 5.01 software (GraphPad Software Inc., USA). Data were mainly exhibited as mean ± standard deviation, median (25th - 75th quantile) or count (percentage). Comparison between two groups was determined by Wilcoxon rank sum test, while comparison among three groups was determined by Kruskal-Wallis H rank sum test. The value of lncRNA expressions for PSA risk was determined by Receiver operating characteristic (ROC) curve. Correlation was determined by Spearman test. *P* < 0.05 was considered as significant.

## Results

### PCA plot and heatmap analysis of lncRNA and mRNA profiles

**Fig. 2:**
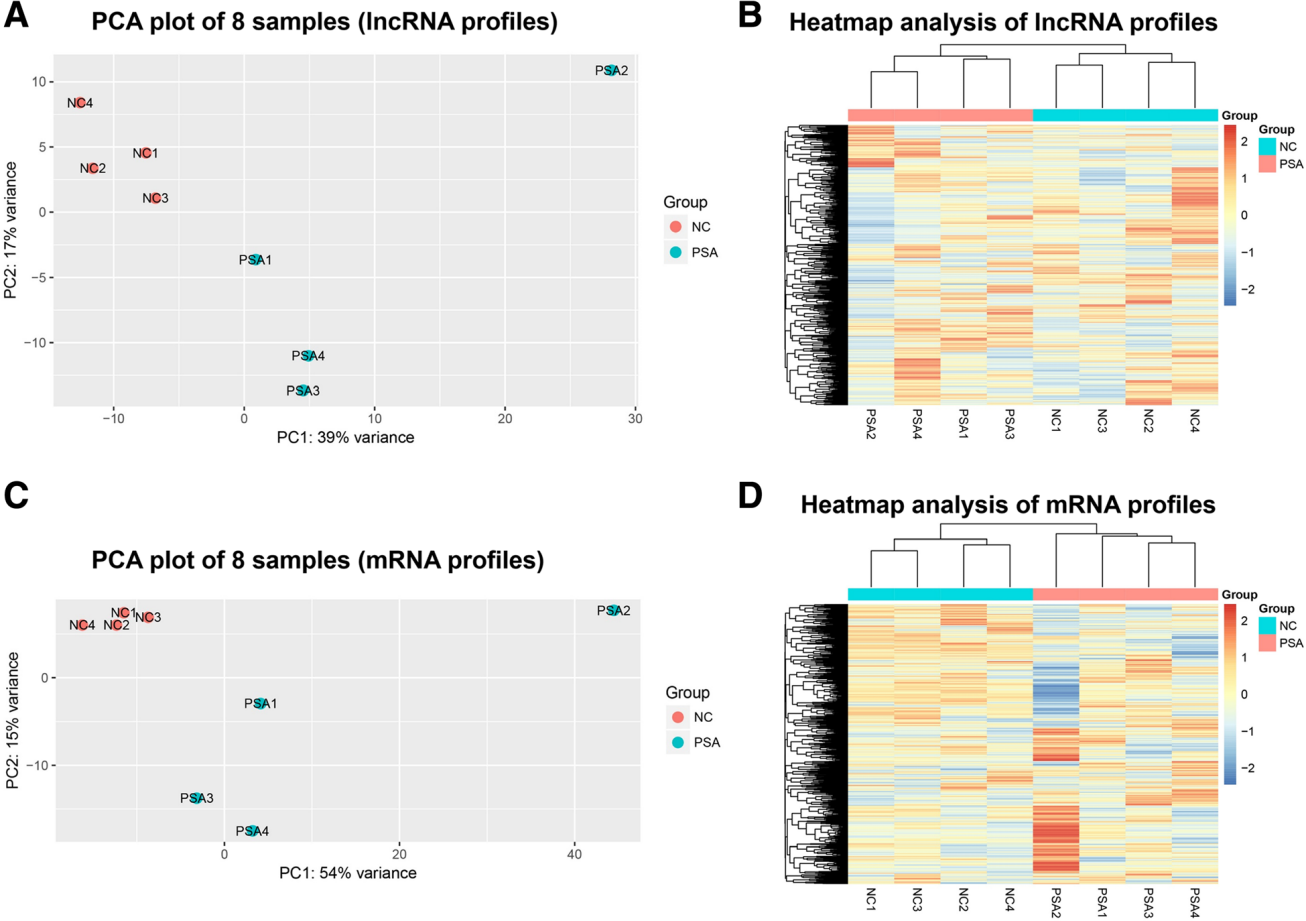
PCA plot and heatmap analysis. PCA plot showed a clear separation between 4 PSA patients and 4 NCs by lncRNA profiles (**a**). The heatmap analysis of lncRNA profiles revealed that lncRNA profiles could differentiate PSA patients from NCs (**b**). PCA plot (**c**) and heatmap analysis (**d**) of mRNAs profiles disclosed that mRNA profiles also clearly segregated PSA patients from NCs. PCA, principal component analysis; *PSA* Psoriatic arthritis, *NCs* Normal controls, *LncRNA* Long non-coding RNA

To determine whether these 8 samples of PSA patients and NCs could be grouped, PCA was performed, which showed a clear segregation between 4 PSA patients and 4 NCs by lncRNA profiles (Fig. 2a). The heatmap analysis of lncRNA profiles showed that lncRNA profiles were able to differentiate PSA patients from NCs (Fig. 2b). PCA plot (Fig. 2c) and heatmap analysis (Fig. 2d) of mRNAs profiles disclosed that mRNA profiles also clearly segregated PSA patients from NCs.

### Volcano plot and heatmap analysis for DELs and DEMs

**Fig. 3:**
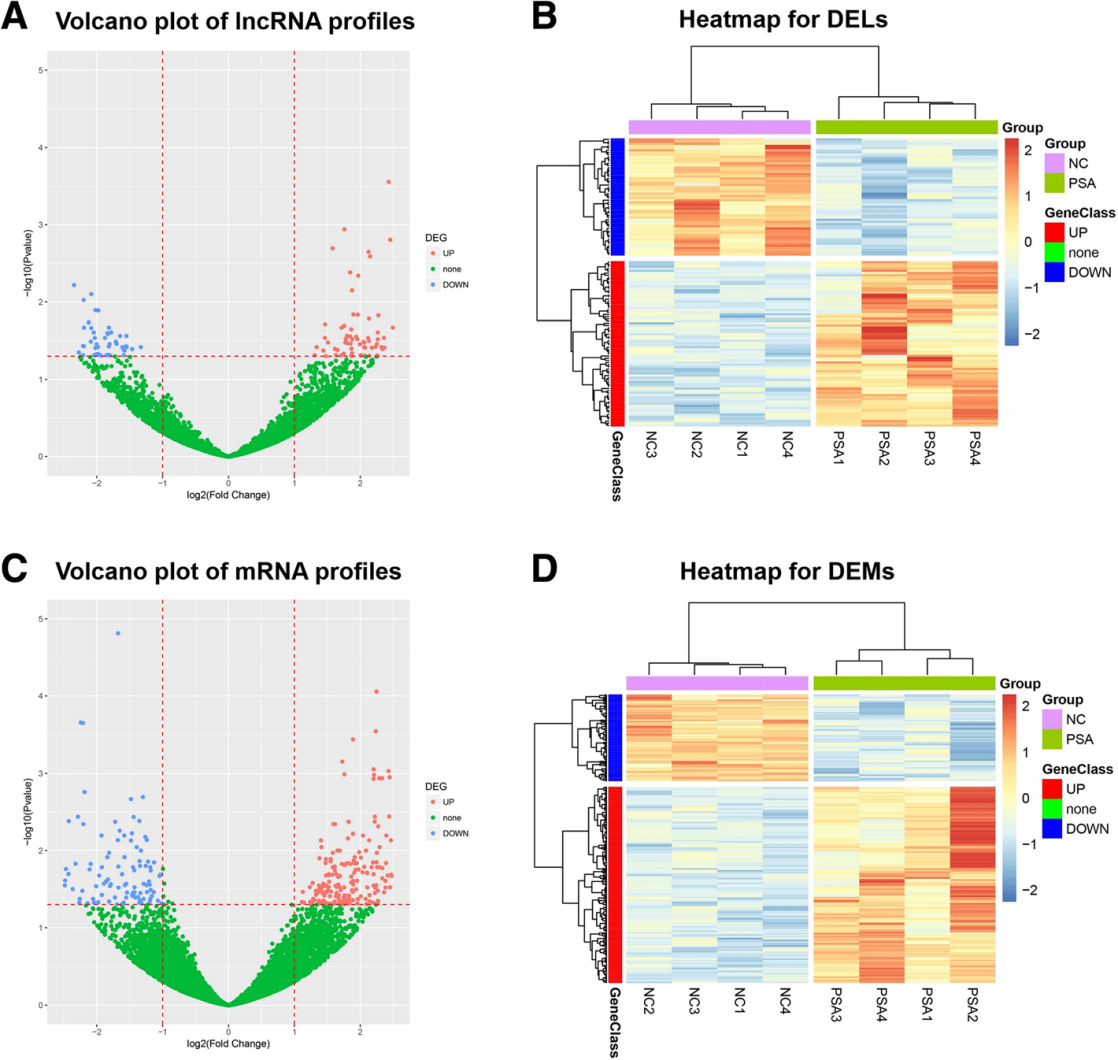
Volcano plot and heatmap analysis. Volcano plot showed that 76 lncRNAs were upregulated and 54 lncRNAs were downregulated in PSA patients compared with NCs (**a**). Heatmap analysis revealed that these DELs were able to separate PSA patients from NCs (**b**). 231 mRNAs were upregulated while 102 mRNAs were downregulated in PSA patients compared with NCs (**c**), and these DEMs could also separate PSA patients from NCs (**d**). lncRNA, long non-coding RNA; PSA, psoriatic arthritis; NCs, normal controls; DELs, differentially expressed lncRNAs; DEMs, differentially expressed mRNAs

A total of 10,079 lncRNAs which were detected in no less than 50% of samples were identified and included into analysis, then 76 remarkably upregulated and 54 remarkably downregulated lncRNAs were found in PSA patients compared with NCs using BH adjusted method (*p* < 0.05 and fold change≥2) (Fig. 3a). And the top 10 upregulated and top 10 downregulated lncRNAs were displayed in Table 1. Heatmap analysis revealed that these DELs could well separate PSA patients from NCs (Fig. 3b). As to DEMs, totally 13,737 mRNAs were identified, among which 231 mRNAs were obviously upregulated while 102 mRNAs were obviously downregulated in PSA patients compared with NCs (Fig. 3c), and these DEMs were also able to separate PSA patients from NCs (Fig. 3d).

**Table 1:** Top 10 upregulated and 10 downregulated lncRNAs in PSA patients

Gene symbol	Gene ID	Location	Log_2_FC	*P* value	Adjusted *P* value	Trend
RP11-701H24.7	ENSG00000271347	Chromosome 15	2.514886	1.42E-08	1.46E-05	Up
RNU12	ENSG00000270022	Chromosome 22	3.596083	1.93E-08	1.62E-05	Up
SNORD3A	ENSG00000263934	Chromosome 17	3.613044	3.39E-08	2.59E-05	Up
RP11–18B3.2	ENSG00000232486	Chromosome 9	2.433375	5.27E-07	0.000278	Up
RNVU1–19	ENSG00000275538	Chromosome 1	2.855135	1.15E-06	0.00052	Up
RP11-707G18.1	ENSG00000278743	Chromosome 12	3.111537	1.76E-06	0.000724	Up
RP11–1250I15.2	ENSG00000278900	Chromosome 5	2.9676	3.00E-06	0.001047	Up
FAM35CP	ENSG00000259096	Chromosome 14	1.758637	3.53E-06	0.001143	Up
TAS2R63P	ENSG00000256019	Chromosome 12	2.454095	5.50E-06	0.001564	Up
RP11-519C12.1	ENSG00000259935	Chromosome 15	1.580232	7.75E-06	0.002017	Up
TRAV1–2	ENSG00000256553	Chromosome 14	−2.99834	4.30E-08	3.05E-05	Down
TRAV1–1	ENSG00000255569	Chromosome 14	−2.70156	4.81E-06	0.001433	Down
TRDV2	ENSG00000211821	Chromosome 14	−2.54527	6.20E-06	0.001711	Down
RASA3-IT1	ENSG00000232487	Chromosome 13	−2.6578	6.94E-06	0.001858	Down
RP11-799B12.2	ENSG00000264924	Chromosome 18	−2.34601	3.27E-05	0.006026	Down
ASMTL-AS1	ENSG00000236017	Chromosome X	−2.08107	5.16E-05	0.007877	Down
C18orf15	ENSG00000279020	Chromosome 18	−2.20011	6.42E-05	0.009423	Down
CTD-2275D10.2	ENSG00000250574	Chromosome 5	−2.01895	9.84E-05	0.01271	Down
RP11-571F15.3	ENSG00000233668	Chromosome 9	−1.97609	0.000101	0.012913	Down
CTC-265F19.1	ENSG00000267749	Chromosome 19	−2.65577	0.000108	0.013541	Down

*LncrRNA* Long non-coding RNA, *PSA* Psoriatic arthritis, *FC* Fold change

### GO and KEGG enrichment analyses for DELs and DEMs

**Fig. 4:**
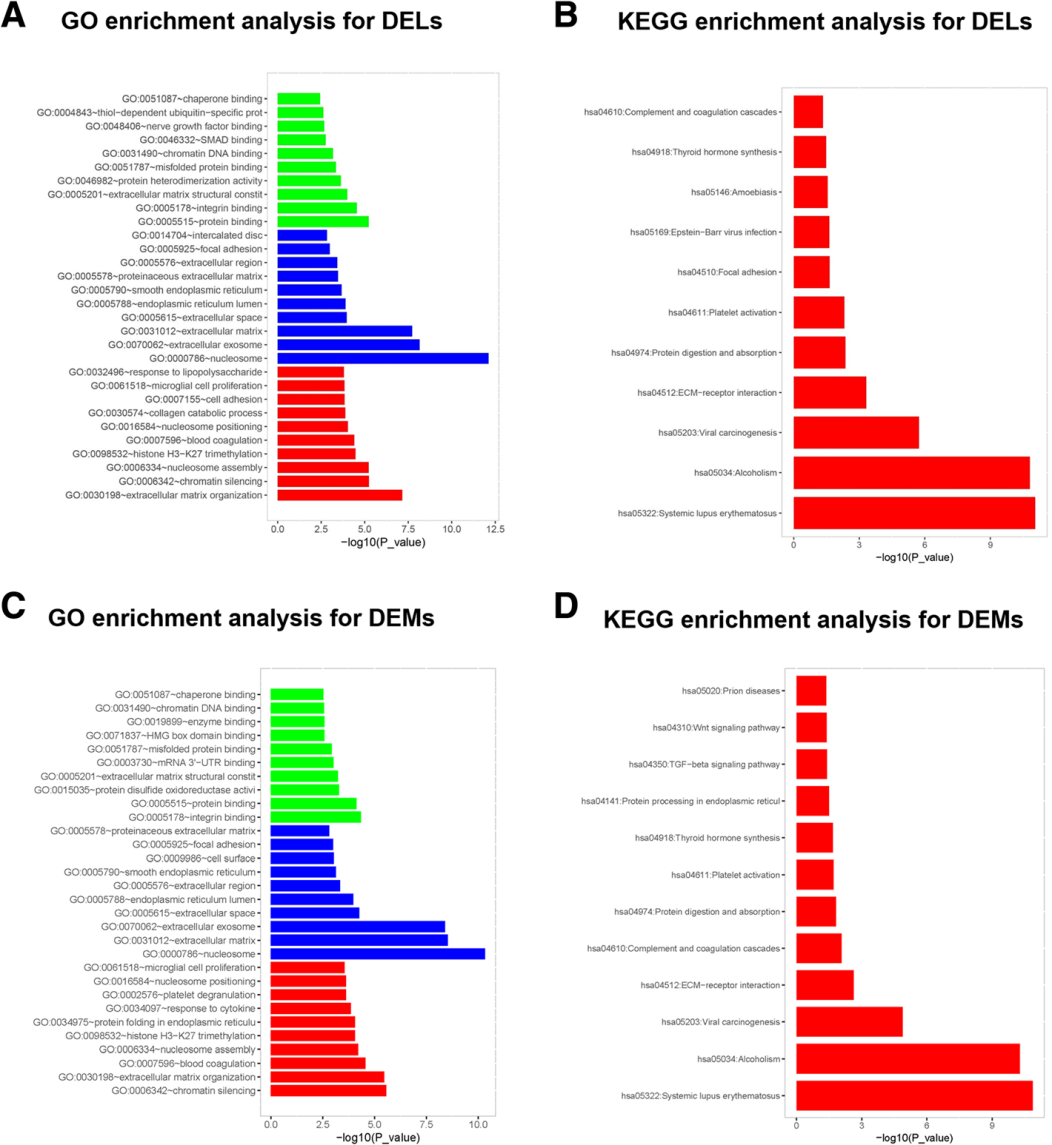
GO and KEGG enrichment analyses. GO enrichment analysis for DELs revealed that DELs were mainly linked to nucleosome, extracellular exosome and extracellular matrix (**a**), KEGG enrichment analyses for DELs showed that DELs were mostly enriched in SLE, alcoholism and viral carcinogenesis (**b**). As for DEMs, their enrichment analysis results were consistent with DELs (**c**, **d**). *GO* Gene Ontology; *KEGG* Kyoko Encyclopedia of Genes and Genomes, *DELs* Differentially expressed lncRNAs, *SLE* Systemic lupus erythematosus, *DEMs* Differentially expressed mRNAs

The enrichment analyses are commonly used to illuminate the molecular functions, biological processes, cellular components and signaling pathways that DELs and DEMs might be implicated. In the current study, GO enrichment analysis for DELs showed that DELs were mostly associated with nucleosome, extracellular exosome and extracellular matrix (Fig. 4a), KEGG enrichment analyses for DELs revealed that DELs were mainly enriched in SLE, alcoholism and viral carcinogenesis (Fig. 4b). As for DEMs, their enrichment analysis results were consistent with that of DELs (Figs. 3c, 4d). These results illustrated the potential pathways and functions that the DELs and DEMs in PSA were involved.

### Regulatory networks of DELs

**Fig. 5:**
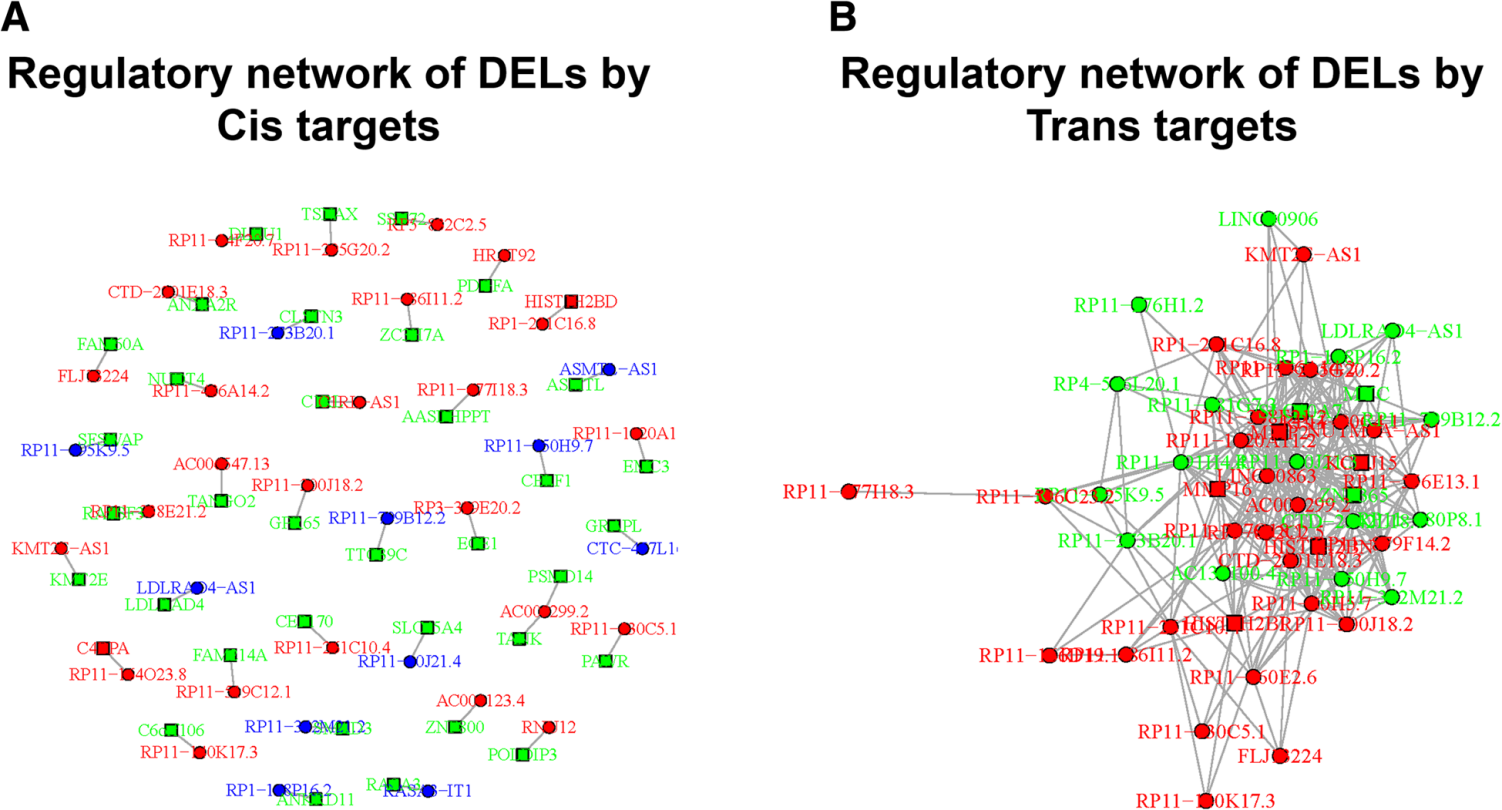
Regulatory networks. Regulatory networks of DELs with their target mRNAs were drawn, including cis targets (**a**) and trans targets (**b**). The dots stood for the DELs, the squares stood for mRNAs; upregulated genes were colored red, downregulated genes were colored blue, and the regulation insignificant genes were colored green. *DELs* Differentially expressed lncRNAs

In order to better understand the functions of DELs in pathogenesis of PSA, the regulatory networks of DELs with their target mRNAs were drawn, including cis targets (Fig. 5a) and trans targets (Fig. 5b). Some DELs have no target mRNA (not displayed in the networks), while other DELs directly regulated one or more target mRNAs. The dots represented the DELs, the squares represented mRNAs. Upregulated genes were colored red, downregulated genes were colored blue, and the regulation insignificant genes were colored green.

### Characteristics of 93 PSA patients in validation stage

To explore potential values of 5 candidate lncRNAs in predicting disease risk and activity of PSA, 93 PSA patients were recruited for further validation, and the characteristics of these PSA patients were displayed in Table 2. Mean age was 52.2 ± 14.7 years, numbers of males and females were 60 (64.5%) and 33 (35.5%), respectively (Table 2). Besides, affected BSA and PASI score were 18.9 ± 7.4% and 7.0 ± 2.8, respectively. CRP and ESR levels were 1.9 (0.5–10.8) mg/l and 18 (8–39) mm/h. As to SJC and TJC, they were 2 (1–4) joints and 3 (1–6) joints. Other clinical features were displayed in Table 2.

**Table 2:** Characteristics of PSA patients in qPCR validation stage

Parameters	PSA patients (*N* = 93)
Age (years)	52.2 ± 14.7
Gender (n/%)	
Male	60 (64.5)
Female	33 (35.5)
Family history of PSA (*n*/%)	
Yes	21 (22.6)
No	72 (77.4)
Disease duration of psoriasis (years)	12 (6–24)
Disease duration of arthritis (years)	3 (1–7)
Psoriasis subtype (*n*/%)	
Vulgaris	85 (91.4)
Others	8 (8.6)
Arthritis subtype (*n*/%)	
Peripheral	72 (77.4)
Axial	13 (14.0)
Mixed	8 (8.6)
Affected BSA (%)	18.9 ± 7.4
PASI score	7.0 ± 2.8
CRP (mg/l)	1.9 (0.5–10.8)
ESR (mm/h)	18 (8–39)
SJC (joints)	2 (1–4)
TJC (joints)	3 (1–6)
Pain VAS score	5.0 ± 2.6
PGA score	5.1 ± 1.4

Data were presented as mean value±standard deviation, median value (25th–75th quantile) or count (percentage). *PSA* Psoriatic arthritis, *qPCR*, quantitative polymerase chain reaction, *BSA*, Body surface area, *PASI* Psoriasis area and severity index, *CRP* C-reactive protein, *ESR* Erythrocyte sedimentation rate, *SJC* Swollen joint count, *TJC* Tender joint count, *VAS* Visual analogue scale, *PGA* Patients’ global assessment

### Comparison of candidate lncRNA expressions between 93 PSA patients and 93 NCs2

**Fig. 6:**
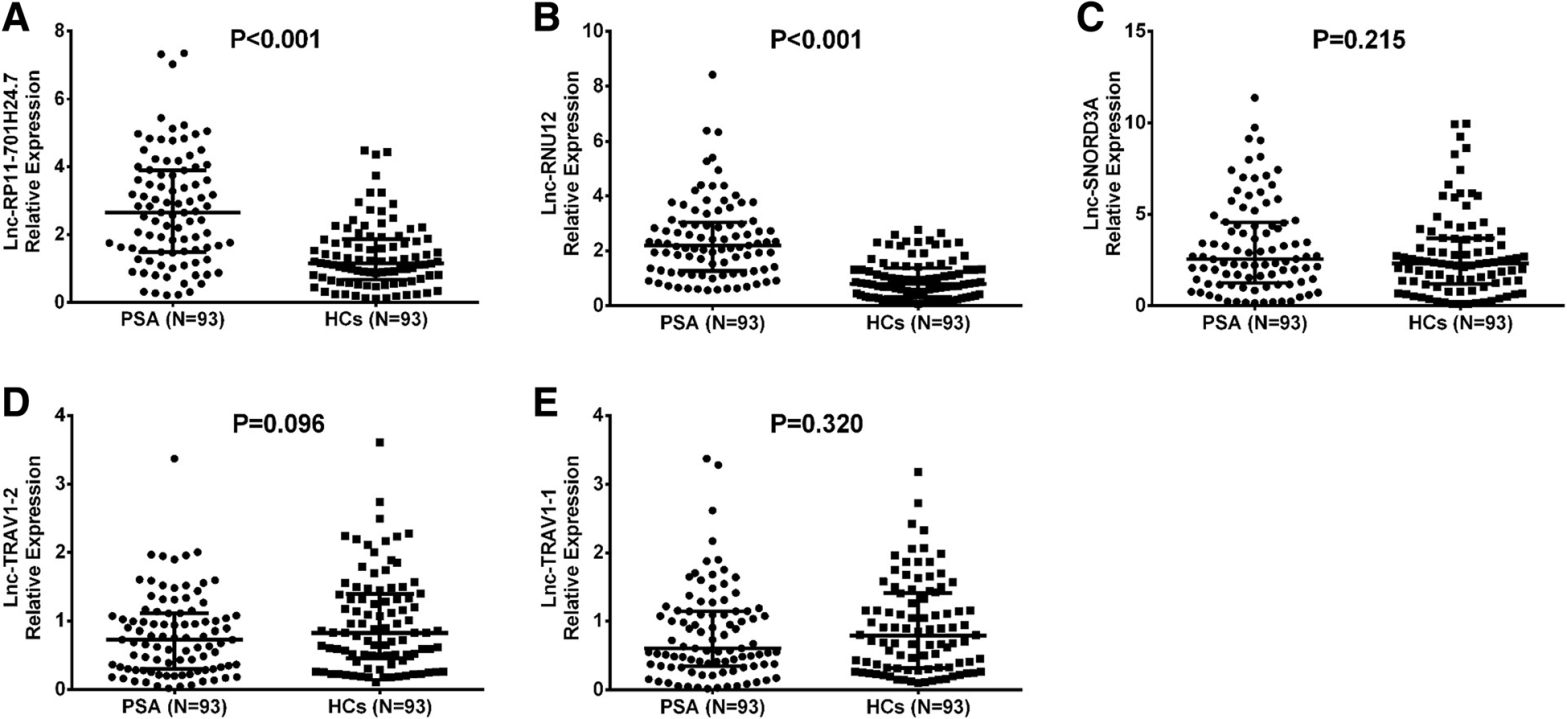
Comparison of candidate lncRNA levels between PSA patients and NCs. Both lnc-RP11-701H24.7 (**a**) and lnc-RNU12 (**b**) levels were elevated in PSA patients compared to NCs, whereas lnc-SNORD3A (**c**), lnc-TRAV1–2 (**d**) and lnc-TRAV1–1 (**e**) levels between two groups were similar. Comparison between two groups was determined by Wilcoxon rank sum test. *P* < 0.05 was considered as significant. *LncRNA*, Long non-coding RNA; *PSA* Psoriatic arthritis; *NCs* Normal controls

3 top upregulated lncRNAs and 2 top downregulated lncRNAs were chosen for further validation in 93 PSA patients and 93 NCs. qPCR assay disclosed that both lnc-RP11-701H24.7 (*P* < 0.001, Fig. 6a) and lnc-RNU12 (*P* < 0.001, Fig. 6b) levels were increased in PSA patients compared with NCs, while lnc-SNORD3A (*P* = 0.215, Fig. 6c), lnc-TRAV1–2 (*P* = 0.096, Fig. 6d) and lnc-TRAV1–1 (*P* = 0.320, Fig. 6e) expressions between two groups were similar.

### ROC curves of lnc-RP11-701H24.7 and lnc-RNU12 for predicting PSA risk

**Fig. 7:**
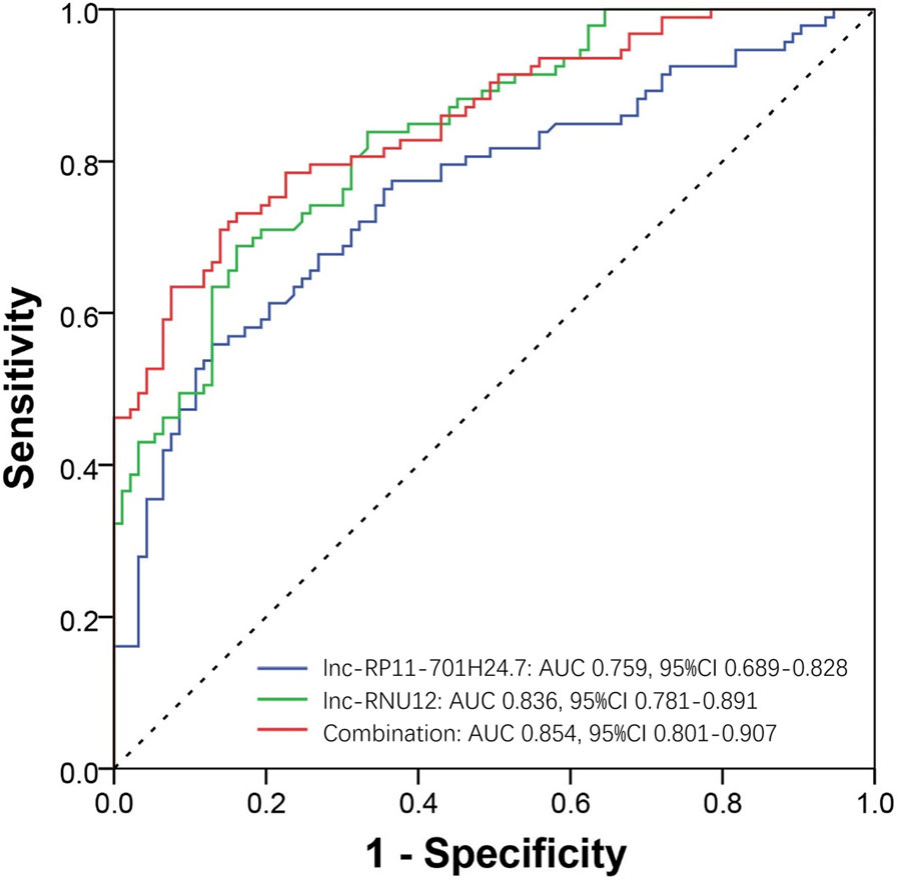
ROC curves. ROC curves revealed that lnc-RP11-701H24.7, lnc-RNU12 and their combination disclosed good predicting values for PSA risk with AUCs of 0.759 (95% CI: 0.689–0.828), 0.836 (95% CI: 0.781–0.891) and 0.854 (95% CI: 0.801–0.907), respectively. ROC curves were drawn to assess the value of lncRNA expressions for PSA risk. *ROC* Receiver operating characteristic, *PSA* Psoriatic arthritis, *AUCs* Area under curves

ROC curves were further drawn to assess the values of lnc-RP11-701H24.7 and lnc-RNU12 in predicting PSA risk, which showed that lnc-RP11-701H24.7, lnc-RNU12 and their combination disclosed good predicting values for PSA risk with AUCs of 0.759 (95% CI: 0.689–0.828), 0.836 (95% CI: 0.781–0.891) and 0.854 (95% CI: 0.801–0.907), respectively (Fig. 7).

### Associations of 5 candidate lncRNAs with characteristics of PSA patients

Kruskal-Wallis H rank sum test, Wilcoxon rank sum test or Spearman test was applied for evaluating associations of 5 candidate lncRNAs with patients’ features, which showed that lnc-RP11-701H24.7 was positively associated with ESR (*P* = 0.049), SJC (*P* = 0.007), TJC (*P* = 0.009) and pain VAS score (*P* = 0.021). Lnc-RNU12 was positively correlated with PASI score (*P* = 0.039), CRP (*P* = 0.024) and PGA score (*P* = 0.011), indicating that both lnc-RP11-701H24.7 and lnc-RNU12 were positively correlated with inflammation level and disease activity of PSA (Table 3**,** Table 4). Meanwhile, lnc-RP11-701H24.7 expression was increased in male patients compared with female patients (*P* = 0.029, Table 3), and lnc-TRAV1–2 was negatively associated with PASI score (*P* = 0.006) as well as PGA score (P = 0.029, Table 4). No associations of the 5 candidate lncRNAs expressions with other features of PSA patients were observed (all *P* > 0.05).

**Table 3:** Correlation of validated lncRNAs with PSA patients’ characteristics (discontinuous variables)

Parameters	lnc-RP11-701H24.7	lnc-RNU12	lnc-SNORD3A	lnc-TRAV1–2	lnc-TRAV1–1
Gender					
Male	3.03 (1.62–4.18)	2.03 (1.23–2.72)	2.54 (1.18–5.03)	0.63 (0.28–1.10)	0.52 (0.29–1.13)
Female	1.92 (0.88–2.94)	2.32 (1.32–3.48)	2.68 (1.50–4.47)	0.78 (0.46–1.14)	0.95 (0.51–1.24)
*P* value	0.029	0.205	0.930	0.312	0.083
Family history of PSA					
Yes	2.65 (1.84–4.04)	2.03 (1.21–2.88)	2.55 (1.29–3.84)	0.76 (0.36–1.24)	0.91 (0.38–1.30)
No	2.59 (1.32–3.76)	2.28 (1.33–3.39)	2.60 (1.23–4.87)	0.71 (0.29–1.08)	0.56 (0.34–1.13)
*P* value	0.517	0.255	0.474	0.762	0.299
Psoriasis subtype					
Vulgaris	2.68 (1.52–3.90)	2.27 (1.25–3.04)	2.55 (1.38–4.55)	0.76 (0.30–1.12)	0.64 (0.35–1.15)
Others	1.80 (1.19–3.68)	1.81 (1.30–3.28)	2.59 (0.47–7.88)	0.53 (0.26–1.06)	0.43 (0.34–0.64)
*P* value	0.351	0.547	0.984	0.547	0.244
Arthritis subtype					
Peripheral	2.66 (1.56–3.97)	2.23 (1.53–2.98)	2.47 (1.18–4.56)	0.62 (0.28–1.03)	0.64 (0.38–1.21)
Axial	1.92 (0.94–3.51)	2.26 (0.90–3.28)	2.68 (1.31–4.80)	0.76 (0.56–1.40)	0.64 (0.34–1.06)
Mixed	3.05 (0.98–4.85)	1.79 (0.99–3.81)	3.26 (2.08–5.48)	1.10 (0.97–1.57)	0.47 (0.24–0.88)
*P* value	0.461	0.766	0.718	0.056	0.522

Data were presented as median value (25th–75th quantile). Comparison was determined by Kruskal-Wallis H rank sum test or Wilcoxon rank sum test. *P* < 0.05 was considered as significant. *LncRNA*, Long non-coding RNA, *PSA* Psoriatic arthritis

**Table 4:** Correlation of validated lncRNAs with PSA patients’ characteristics (continuous variables)

	lnc-RP11-701H24.7	lnc-RNU12	lnc-SNORD3A	lnc-TRAV1–2	lnc-TRAV1–1
Age					
correlation coefficient r	0.103	0.014	−0.036	0.033	0.050
*P* value	0.326	0.893	0.735	0.750	0.635
Disease duration of psoriasis					
correlation coefficient r	−0.029	− 0.019	− 0.024	− 0.021	0.137
*P* value	0.789	0.857	0.822	0.844	0.198
Disease duration of arthritis					
correlation coefficient r	0.063	0.046	0.009	−0.056	0.151
* P* value	0.562	0.675	0.931	0.604	0.161
Affected BSA					
correlation coefficient r	−0.077	−0.103	−0.097	− 0.054	0.091
*P* value	0.465	0.326	0.354	0.605	0.387
PASI score					
correlation coefficient r	0.005	0.214	−0.071	−0.281	0.091
*P* value	0.963	0.039	0.496	0.006	0.383
CRP					
correlation coefficient r	0.172	0.269	0.055	−0.189	−0.035
*P* value	0.155	0.024	0.648	0.118	0.772
ESR					
correlation coefficient r	0.242	0.115	−0.012	−0.098	−0.021
P value	0.049	0.353	0.925	0.432	0.867
SJC					
correlation coefficient r	0.284	0.198	0.048	0.065	−0.061
*P* value	0.007	0.061	0.656	0.540	0.569
TJC					
correlation coefficient r	0.273	0.199	−0.085	−0.025	0.066
*P* value	0.009	0.060	0.426	0.819	0.535
Pain VAS score					
correlation coefficient r	0.239	0.133	−0.011	0.002	0.025
*P* value	0.021	0.202	0.916	0.984	0.811
PGA score					
correlation coefficient r	0.075	0.263	−0.033	−0.227	0.063
*P* value	0.475	0.011	0.756	0.029	0.547

Data were presented as correlation coefficient r and *P* value. Correlation was determined by Spearman test. *P* < 0.05 was considered as significant. *LncRNA* Long non-coding RNA, *PSA* Psoriatic arthritis, *BSA* Body surface area, *PASI* Psoriasis area and severity index, *CRP* C-reactive protein, *ESR* Erythrocyte sedimentation rate, *SJC* Swollen joint count, *TJC* Tender joint count, *VAS* Visual analogue scale, *PGA* Patients’ global assessment

## Discussion

LncRNA, a group of RNAs that is described in 2002 for the first time, presents with several characteristics, including poor conservation, tissue specificity and so on [7, 18, 19]. LncRNAs exist in almost every branch of life and are involved various important biological activities such as genomic imprinting, cell differentiation and organogenesis [20, 21]. LncRNAs regulating gene expressions are mainly through three different ways: transcription regulation, post-transcription regulation or epigenetic regulation [22]. For transcription regulation, lncRNAs are able to regulate different transcription processes such as modulate transcription factor activity, mediate RNA polymerase (RNAP) II activity and regulate the functions of RNA binding protein. For post-transcription regulation, lncRNAs also modulate various post-transcriptional processes, such as splicing, editing, transport, translation and degradation. Besides, lncRNAs are also involved epigenetic regulations such as imprinting and X-chromosome inactivation by recruiting chromatin remodeling complexes to specific genomic loci [23, 24]. In recent years, the role of lncRNAs in a number of diseases have been intensively investigated, especially in complicated diseases such as cancers, CAD and autoimmune diseases [18]. Taken psoriasis as an example, a study explores the lncRNA expression profiles in skin sample of psoriasis patients, which identifies 1080 novel differentially expressed lncRNAs in psoriasis patients compared with controls, further analyses revealed that these dysregulated lncRNAs are mostly enriched in biosynthesis of unsaturated fatty acid and cytokine activity [25]. However, the implications of lncRNA expression profiles in PSA are still obscure.

PSA is a systemic inflammatory disease that both genetic and environmental factors play crucial roles in the etiology of this disease [5]. Since lncRNA expression profiles are reported to be paramount in etiopathologies of autoimmune diseases such as SLE, IBD and RA, exploring lncRNA expression profiles of PSA might also be able to provide new insights for PSA pathogenesis and find novel biomarkers for predicting PSA risk and activity [9–11]. Whereas relevant study is rarely found until now, with only one study reported [12]. In this previous study, lncRNA expression profiles in PBMC of 10 PSA patients are analyzed by microarray, which discovers 259 DELs in PSA patients compared with HCs, further analyses reveal that these DELs are implicated PSA-related signaling pathways such as immune response, glycolipid metabolism, bone remodeling and type 1 interferon. Subsequently, 7 lncRNAs (TRIM55–1, EPB41L4A-AS1, LINC00657, LINC00909, RP11-539 L10.3, LA16c-360H6.3 and LUCAT1) are selected for validation in 20 PSA patients and 20 HCs, which discloses that LA16c-360H6.3 is upregulated while the rest of 6 lncRNAs are downregulated in PSA patients compared with HCs [12]. However, this study uses microarray to detect DELs rather than RNA sequencing. What’s more, the sample size for validation is small, with only 20 samples in each group, which also decreases statistical power. Considering that no more studies are reported to explore the role of lncRNA expression profiles in PSA, and the potential pathways or mechanisms that lncRNA expression profiles might be involved are largely unelucidated, we conducted the current study by RNA sequencing, which discovered 130 DELs in PSA patients compared to NCs, among which 76 lncRNAs were upregulated while 54 lncRNAs were downregulated. The number of DELs in our study was smaller than the previous study, which might be due to the following reasons: (1) our study discarded those lncRNAs that were discovered in less than 50% samples, whereas the previous study does not. (2) Our study utilized RNA sequencing to detect DELs, while the reported study uses microarray to detect DELs, which might cause differences. (3) DELs in our study must meet an adjusted *P* value < 0.05 (BH adjusted method) and a fold change≥2.0, while BH adjusted method is not used in their study. Taken together, the DELs in the current study were relatively fewer than that of reported study. In addition, we compared our data-set with that previous study, discovering that 1 DEL and 41 DEMs in our data-set were overlapped with that previous study, indicating that the remaining 129 DELs and 292 DEMS in our study were first identified.

The enrichment analyses are prevalent approaches to illuminate the molecular functions, biological processes, cellular components and signaling pathways that differentially expressed genes might be implicated [26, 27]. In the current study, enrichment analyses disclosed that DELs and DEMs were mostly associated with nucleosome, extracellular exosome and extracellular matrix, and the top enriched pathways were SLE, alcoholism and viral carcinogenesis. The possible explanations for the results might be that: for nucleosome, it is reported that its loss contributes to macrophage activities, indicating that DELs involved PSA might be through regulating DEMs then affecting the numbers of nucleosome thereby activating macrophages [28]. For extracellular exosome, it is discovered to induce inflammatory immune response in a couple of autoimmune diseases such as SLE and RA, and extracellular exosome in PSA patients is able to stimulate osteoclast differentiation, suggesting that DELs implicated PSA might also be through regulating DEMs then modulating exosome thereby mediated osteoclast differentiation [29, 30]. For extracellular matrix, it serves important functions to cell adhesion, cell-to-cell communication and cell differentiation, and the dysregulated degradation of extracellular matrix is associated with a number of diseases such as osteoarthritis and PSA, therefore DELs involved PSA might also be through regulating DEMs then mediating degradation of extracellular matrix [31, 32]. For SLE, it closely correlates with autoimmunity and inflammation, which are similar to PSA. As for alcoholism and viral carcinogenesis, their associations with PSA need further investigation.

The disease manifestations of PSA are sometimes varied from patients to patients, which make the diagnosis difficult. To explore if there are DELs that could be served as biomarkers for disease risk, inflammation or activity of PSA, 3 top upregulated and 2 downregulated lncRNAs were selected and their expressions were validated in 93 PSA patients and 93 NCs by qPCR assay, which revealed that both lnc-RP11-701H24.7 and lnc-RNU12 expressions were increased in PSA patients compared with NCs, and they disclosed good predictive values for PSA risk with high AUCs. Besides, lnc-RP11-701H24.7 and lnc-RNU12 were also positively associated with inflammation level and disease activity of PSA. The possible reasons might be that: both lnc-RP11-701H24.7 and lnc-RNU12 might be implicated PSA development and progress through (i) epigenetic regulations such as histone and DNA methylation. (ii) Mediating gene transcription; (iii) regulating its target genes such as COL1A2, RNU12 and MAP1A. However, the in-depth mechanisms of lnc-RP11-701H24.7 and lnc-RNU12 being implicated PSA pathogenesis need further investigation [33]. In addition, we also observed that lnc-TRAV1–2 was negatively associated with PASI score and PGA score, indicating that lnc-TRAV1–2 might be also correlated with decreased disease activity of PSA. In short, it was the first study to discover specific lncRNAs that might be regarded as biomarkers for PSA risk and activity, which might remarkably promote the early diagnosis and contribute to discovering novel approaches for improving long-term outcomes of PSA patients.

There were some limitations in the current study. First, the study did not evaluate the treatment response of PSA patients, so the role of lncRNA expression profiles in treatment response and long-term outcome of PSA patients were not investigated. Second, most patients in this study were from East China, which might bring in selection bias. Third, although we discovered the positive correlations of lnc-RP11-701H24.7 and lnc-RNU12 with disease risk and activity, the detailed mechanisms of these two lncRNAs in development and progression of PSA remained unclear, which needed to be further explored in our future work.

## Conclusion

In summary, our study facilitates comprehensive understanding of lncRNA expression profiles in PSA development and progression, and discovers that lnc-RP11-701H24.7 and lnc-RNU12 might be served as novel biomarkers for PSA risk and activity.

## Additional file

Additional file 1: **Table S1.** Primers used in qPCR validation. (DOCX 15 kb)

## Data Availability

All data generated or analysed during this study are included in this published article.

## Abbreviations

BH: Benjamini & Hochberg
BSA: body surface area
CAD: cardiovascular disease
CASPAR: Classification of Psoriatic Arthritis
CRP: C-reactive protein
DELs: differentially expressed lncRNAs
DEMs: differentially expressed mRNAs
ESR: erythrocyte sedimentation rate
GAPDH: phosphoglyceraldehyde dehydrogenase
GO: Gene Ontology
HCs: healthy controls
IBD: inflammatory bowel disease
KEGG: Kyoko Encyclopedia of Genes and Genomes
lncRNA: Long non-coding RNA
NCs: normal controls
PASI: psoriasis area and severity index
PBMC: peripheral blood mononuclear cells
PCA: Principal component analysis
PGA: patients’ global assessment
PSA: Psoriatic arthritis
qPCR: quantitative polymerase chain reaction
RA: rheumatic arthritis
ROC: Receiver operating characteristic
SJC: swollen joint count
SLE: systemic lupus erythematosus
TJC: tender joint count
VAS: Pain visual analogue scale
