# Supplementary material for: Comprehensive analyses of long non-coding RNA expression profiles by RNA sequencing and exploration of their potency as biomarkers in psoriatic arthritis patients
Source: BMC Immunol. 2019 Aug 7;20:28. doi: 10.1186/s12865-019-0297-9 (PMC6686418; doi:10.1186/s12865-019-0297-9)
Supplement: Supplementary file 1 — Table S1. Primers used in qPCR validation. (DOCX 15 kb) [file 12865_2019_297_MOESM1_ESM.docx]

**Supplementary Table 1.** Primers used in qPCR validation.

| Gene | Forward Primer | Reverse Primer |
| --- | --- | --- |
| lnc-RP11-701H24.7 | 5’ AGAAAATGACATGGGAAGCGT 3’ | 5’ ACACTGTATCCTCAAATGCACA 3’ |
| lnc-RNU12 | 5’ CTTGTTGGTAGGTGGTGAATGC 3’ | 5’ TGAAGCACAGAGAAGCGGTTAT 3’ |
| lnc-SNORD3A | 5’ CCACGAGGAAGAGAGGTAGC 3’ | 5’ CCCAATACGGAGAGAAGAACGA 3’ |
| lnc-TRAV1-2 | 5’ ATGGGAGGCACTACAGGACAA 3’ | 5’ GCACAGAGGTAAGAGGCAGAG 3’ |
| lnc-TRAV1-1 | 5’ GGAAGGAGCCATTGTCCAGATAA 3’ | 5’ ACCATAACTATCAGAGCGACTAAGG 3’ |
| GAPDH | 5’ GAGTCCACTGGCGTCTTCAC 3’ | 5’ ATCTTGAGGCTGTTGTCATACTTCT 3’ |

qPCR, quantitative polymerase chain reaction.
